# Antigen-Specificity in the Thymic Development and Peripheral Activity of CD4^+^FOXP3^+^ T Regulatory Cells

**DOI:** 10.3389/fimmu.2018.01701

**Published:** 2018-07-23

**Authors:** Jelka Pohar, Quentin Simon, Simon Fillatreau

**Affiliations:** ^1^Institut Necker-Enfants Malades, INSERM U1151-CNRS UMR 8253, Paris, France; ^2^Faculté de Médecine, Université Paris Descartes, Sorbonne Paris Cité, Paris, France; ^3^AP-HP, Hôpital Necker Enfants Malades, Paris, France

**Keywords:** T regulatory cells, T cell, Tconv, Foxp3, autoimmunity, allergy, Goodpasture’s disease, experimental autoimmune encephalomyelitis

## Abstract

CD4^+^Foxp3^+^ T regulatory cells (Treg) are essential for the life of the organism, in particular because they protect the host against its own autoaggressive CD4^+^Foxp3^−^ T lymphocytes (Tconv). Treg distinctively suppress autoaggressive immunity while permitting efficient defense against infectious diseases. This split effect indicates that Treg activity is controlled in an antigen-specific manner. This specificity is achieved first by the formation of the Treg repertoire during their development, and second by their activation in the periphery. This review presents novel information on the antigen-specificity of Treg development in the thymus, and Treg function in the periphery. These aspects have so far remained imprecisely understood due to the lack of knowledge of the actual antigens recognized by Treg during the different steps of their life, so that most previous studies have been performed using artificial antigens. However, recent studies identified some antigens mediating the positive selection of autoreactive Treg in the thymus, and the function of Treg in the periphery in autoimmune and allergic disorders. These investigations emphasized the remarkable specificity of Treg development and function. Indeed, the development of autoreactive Treg in the thymus was found to be mediated by single autoantigens, so that the absence of one antigen led to a dramatic loss of Treg reacting toward that antigen. The specificity of Treg development is important because the constitution of the Treg repertoire, and especially the presence of holes in this repertoire, was found to crucially influence human immunopathology. Indeed, it was found that the development of human immunopathology was permitted by the lack of Treg against the antigens driving the autoimmune or allergic T cell responses rather than by the impairment of Treg activation or function. The specificity of Treg suppression in the periphery is therefore intimately associated with the mechanisms shaping the formation of the Treg repertoire during their development. This novel information refines significantly our understanding of the antigen-specificity of Treg protective function, which is required to envision how these cells distinctively regulate unwanted immune responses as well as for the development of appropriate approaches to optimally harness them therapeutically in autoimmune, malignant, and infectious diseases.

## Introduction

CD4^+^Foxp3^+^ T regulatory cells (Treg) are required throughout life to maintain a healthy immune system because conventional CD4^+^ T cells (Tconv) pose a permanent threat of deadly autoimmune attack if inappropriately controlled (1, 2). In human, this is illustrated by the early development of the severe immune dysregulation polyendocrinopathy, enteropathy X-linked (IPEX) syndrome in patients lacking Treg due to a *FOXP3* gene deficiency (3). IPEX can be fatal within the first year of life, and is often associated with insulin-dependent diabetes mellitus, enteropathy, eczema, thrombocytopenia, anemia, as well as cachexia (3). The generation of Treg occurs both in the thymus (thymus-derived tTreg account for around 95% of peripheral Treg), and in the periphery including at mucosal surfaces where some Tconv convert into peripherally generated Treg (pTreg) (4, 5). These separate ontogenic pathways might account for the existence of Treg reacting toward distinct categories of antigens including self-antigens (6–8), innocuous environmental antigens, or pathogens (9–12). Both tTreg and pTreg are required for the maintenance of immune homeostasis (13, 14).

There is clear indication that the generation of Treg is decisively influenced by the reactivity of their T cell receptor (TCR) for antigen (4). In mice with monoclonal T cells, the differentiation of tTreg is only observed with some TCR (15), and the TCR repertoires of Tconv, tTreg, and pTreg are largely different (16–19). This raises the question of the mode of antigen recognition by the TCR that is associated with the commitment of T cell precursors to the tTreg lineage. An initial study showed that the ectopic expression of a model antigen in the thymus resulted in the commitment of T cell precursors carrying a transgenic TCR of high affinity for this antigen (but not of those bearing a TCR of lower affinity) into tTreg (20). Since multiple tissue-restricted antigens are promiscuously expressed in the thymus (21, 22), this led to the concept that the recognition of promiscuously expressed self-antigens *via* TCR–major histocompatibility complex (MHC-II) interactions of high affinity drove autoreactive tTreg development. However, other data obtained using distinct systems for the expression of transgenic antigens did not agree with this model (23). Furthermore, studies on the negative selection of T cell progenitors have shown that preventing the negative selection of highly autoreactive T cells in the thymus was not always associated with their diversion into the tTreg lineage (24). These conflicting observations led to some uncertainty in the field. TCR signals are also important for the function of Treg in the periphery (25, 26), and the modulation of TCR signaling has been used to increase or decrease their regulatory activity (24, 27). However, the relevance of antigen-specificity for the function of Treg in human disease is poorly understood, largely due to the lack of knowledge of the antigen pertinent for Treg function in human disease.

It was therefore important to use novel approaches in order to directly address how “real” endogenous (or exogenous) antigens actually select tTreg, and how antigen-specificity contributes to Treg function in the periphery. Such investigations have for a long time been hampered by the lack of the knowledge of the endogenous antigens recognized by Treg, and the difficulty of identifying them. Recent studies overcame this obstacle and identified physiological agonists recognized by Treg in the thymus, and in the periphery. These recent advances help understanding the relationship between the specificity of tTreg selection in the thymus and the specificity of tTreg-mediated suppression in the periphery, as well as how the antigen-specificity of Treg relates to the prevention or the failure to prevent autoimmune disorders and allergies. This review discusses the role of antigen-specificity in Treg development and function in the light of these recent studies.

## Endogenous Self-Antigens and the Selection of Autoreactive Treg in the Thymus

The role of the thymus in dominant immune tolerance was brought to light from the observation that thymectomy at day 3, but not at day 7, after birth led to the development of fatal autoimmune attacks affecting various tissues (28). This phenomenon is due to the delayed emergence of tTreg relatively to Tconv during development (29). Indeed, the thymus produces almost exclusively Tconv before day 3, so that thymectomy at this time results in a situation of Treg deficiency. As already mentioned, the importance of cognate antigen recognition for the development of tTreg was initially indicated by experiments involving double transgenic mice ectopically expressing the hemagglutinin (HA) from influenza virus in the thymus, and a TCR specific for this antigen in T cells (30). In the thymus, T cell progenitors expressing a transgenic TCR of high affinity for HA gave rise to tTreg while those expressing a specific TCR of lower affinity for this antigen did not, suggesting that the development of tTreg was instructed by TCR interactions of high affinity for their cognate antigen (30). Observations made using an Nur77-eGFP reporter mice, in which the relative level of TCR signaling could be inferred from the level of eGFP expression, also indicated in the context of a wild-type TCR repertoire that Treg underwent stronger TCR signaling than Tconv during their thymic development (31). However, the concept that the autoimmune reactivity of a TCR rules its capacity to instruct Treg development was not supported by data obtained using other transgenic systems, as discussed in other reviews (20, 23). Moreover, some studies of the signaling processes regulating T cell fate decision in the thymus indicated that higher affinity was not always associated with increased tTreg selection (24). It therefore appeared important to identify endogenous antigens mediating tTreg selection, and to characterize the TCR–MHC-II–antigen peptide interaction through which they generated tTreg, in order to refine this model.

Several endogenous antigens implicated in tTreg development have recently been identified. A first study analyzed the development and function of Treg specific for myelin oligodendrocyte glycoprotein (MOG), a minor component of the myelin sheath that is targeted by T cells and autoantibodies in autoimmune diseases of the central nervous system (CNS) such as multiple sclerosis and its animal model experimental autoimmune encephalomyelitis (EAE) (32). After immunization with rodent MOG, C57BL/6 mice develop an EAE driven by MOG-reactive CD4^+^ T cells. This T cell response is dominated by a public TCRβ rearrangement found in both pathogenic Tconv and protective Treg in the CNS of sick mice (33, 34). Using mice carrying this TCRβ as a transgene, it was possible to compare the repertoire and antigen-recognition properties of the TCR carried by disease-relevant MOG-reactive Treg and Tconv, which were taken from the CNS at the peak of EAE, following the characterization of their TCRα chain (34). The analysis of 17 TCR from Treg and 11 TCR from Tconv obtained in such a way showed that Treg expressed TCR of markedly higher functional affinity for MOG than Tconv (34). The TCR from Treg and Tconv also interacted with MOG–MHC-II complexes in a qualitatively different manner (34). This was shown using MOG peptide variants differing from the endogenous MOG peptide sequence by alanine substitutions at various positions. The substitution of a particular histidine by an alanine in this peptide increased the stimulation of all the TCR from MOG-reactive Tconv by about 10-fold while having little effect on the response triggered by the TCR from MOG-reactive Treg. These data suggest that these TCR from Treg and Tconv differently engage the MOG peptide–MHC-II complex, as previously observed for *in vitro* generated Tr1 cells (35). If this is the case and is a general property, it might be possible to identify small molecules that preferentially interfere with antigen recognition by Tconv but not Treg, and *vice versa*. Another difference between these two types of TCR was that the wild-type MOG peptide was among the best agonists for Treg-derived TCR while this was not the case for Tconv-derived TCR, which displayed stronger responses when stimulated with some altered peptides than with the wild-type one (34). Thus, the best agonists for autoreactive tTreg might be self-peptides, while the best agonists for Tconv might be non-self-peptides, as expected from the divergent functions of these cells. The profound loss of responsiveness observed with MOG peptide variants for Treg-derived TCR suggests that Treg might be able to respond efficiently only to their cognate self-peptide, whereas Tconv might be able to react against a broader diversity of non-self sequences.

These TCR from MOG-reactive Treg and Tconv were then exploited to investigate the role of endogenous MOG in the selection of MOG-reactive tTreg (34). Retrogenic mice were prepared by reconstituting irradiated wild-type mice and *Mog*-deficient mice, which did not show any neurological abnormality (36), with hematopoietic stem cells (HSC) genetically engineered to express a single MOG-reactive TCR (37). In retrogenic mice made using wild-type mice as recipients, donor-derived tTreg only emerged when HSC expressed TCR from MOG-reactive Treg, whereas TCR from MOG-reactive Tconv only generated Tconv, confirming the instructive role of the TCR for tTreg development (34). Remarkably, the production of MOG-reactive tTreg was severely reduced when HSC expressing MOG-reactive TCR from Treg were transferred into *Mog*-deficient recipient mice, while the generation of MOG-reactive Tconv was not affected by the absence of MOG (34). Thus, autoreactive tTreg are positively selected on endogenous self-antigen, and the absence of a single endogenous antigen can lead to a severe loss of tTreg positive selection. By contrast, the selection of MOG-reactive Tconv was not affected by the absence of endogenous MOG, implying that MOG-reactive Tconv were positively selected on distinct antigen in the thymus. The high specificity of the Treg positive selection process is surprising given that the TCR recognition of antigen is viewed as degenerate (38, 39). It was therefore unexpected that none of the other antigen–MHC-II complexes available in the thymus could compensate for the absence of MOG. Similar findings were obtained for autoreactive tTreg reactive against the TRPM8 channel-associated factor 3 (TCAF3) antigen (7). This antigen is exclusively expressed in the prostate, and additionally in the thymus in an *Aire*-dependent manner (7). tTreg directed toward this antigen accumulate in the prostate when this organ develops tumor or autoimmune lesions (7). The analysis of genetically modified mice engineered to lack only the recognized TCAF3 epitope revealed that the absence of this single antigen was sufficient to abrogate the positive selection of TCAF3-reactive tTreg. Thus, tTreg development was also mediated by a single endogenous antigen in this case (7). As a third example, tTreg reacting toward the interphotoreceptor retinoid-binding protein (IRBP) might also be selected in a highly specific manner on IRBP because IRBP-specific Treg-mediated suppression was functionally detectable in wild-type mice but not in *Irbp*-deficient mice, suggesting that IRBP-reactive Treg were absent in these mice (40, 41). Altogether, these independent studies demonstrate that autoreactive tTreg are positively selected in an exquisitely specific manner on natural self-antigen through TCR interaction of high affinity. This modality of selection is relevant for the protective function of Treg in the periphery, and the design of antigen-specific Treg immunotherapy. Indeed, in adoptive cell therapy, polyclonal Treg engineered by TCR gene transfer to express TCR of high affinity for MOG (initially cloned from MOG-reactive Treg) almost completely protected recipient mice from EAE, while Treg manufactured with TCR of low affinity for MOG barely had any protective effect (34). The protective efficacy of engineered autoreactive Treg was also proportional to the affinity of their TCR for the target self-antigen in a model of type 1 diabetes (42). This implies that the antigen-reactive TCR has to be carefully chosen to optimize the beneficial effect of Treg engineered by TCR gene transfer in adoptive cell therapy. Treg expressing antigen-reactive TCR with suboptimal affinity for the target antigen might have no effect on the course of the disease.

In the thymus, TCR–antigen interactions of different strengths can lead to the development of distinct tTreg subsets. A study performed using transgenic mice expressing ovalbumin in the thymus, and six TCR reacting toward ovalbumin with different strength, showed that a broad range of TCR affinity varying by 1,000-fold drove tTreg development with the TCR of highest affinity for ovalbumin giving rise to the largest tTreg production (6). Several tTreg subsets were produced including GITR^hi^PD-1^hi^CD25^hi^ and GITR^lo^PD-1^lo^CD25^lo^ tTreg, which also differed by their expression of Helios, ICOS, CD44, and CD62L (43). Both are selected by self-antigens, being present in similar amount in germ-free mice, antigen-free mice [i.e., germ-free mice fed with a minimal antigen-free diet consisting of glucose and amino acids (44)], and mice kept in standard specific-pathogen-free conditions (43). They differ by their affinity for self-antigens: GITR^hi^PD-1^hi^CD25^hi^ Treg express TCR of higher affinity for self-antigens than GITR^lo^PD-1^lo^CD25^lo^ Treg, consistently with their higher expression of CD5 and Nur77. Remarkably, these two Treg subsets display different functions *in vivo*. GITR^hi^PD-1^hi^CD25^hi^ Treg control T and B cell activation in secondary lymphoid organs, while GITR^lo^PD-1^lo^CD25^lo^ Treg protect recipient mice from colitis induced by T cell transfer (43). Thus, the TCR-mediated recognition of antigen in the thymus not only directs the partitioning of T cell precursors along the tTreg fate but also fixes the functional status of these cells.

The role of endogenous antigens as the selecting moieties for autoreactive tTreg relates to the promiscuous expression of multiple self-antigens, which are otherwise restricted to defined tissues, in medullary thymic epithelial cells (mTEC). This promiscuous antigen expression in mTEC occurs partly in an *Aire*-dependent manner (22, 45, 46). AIRE controls the expression of more than 3,900 genes in mTEC providing a source of endogenous antigens for the positive selection of tTreg and the negative selection of Tconv in the thymus (47). The ectopic expression of tissue-restricted antigens in mTEC is strongly regulated. There are about 100,000–200,000 mTEC in a mouse thymus (48), and each tissue-specific antigen is expressed by only 1–3% of them at a given time (49–51). Although *Aire*-dependent antigens are implicated in the partition of some TCR into the Treg compartment, Malchow and colleagues estimated that only 5% of peripheral Treg develop in an *Aire*-dependent manner (22). Such a modest role for AIRE in Treg selection is in keeping with the finding that Treg from *Aire*-deficient mice can control autoreactive Tconv from wild-type mice (22). It is also consistent with the fact that *Aire*-deficient mice on a C57BL/6 background develop only a mild autoimmune phenotype (52) and survive for more than a year, whereas *Foxp3*-deficient mice become moribund within 4 weeks of age (53). Similarly, humans with loss-of-function mutation in *AIRE* develop the disease autoimmune polyendocrinopathy-candidiasis ectodermal dysplasia (APECED) yet these presents with a different clinical profile than IPEX (54). Nonetheless, APECED patients show a modest decrease in Treg (55–57). These experimental and clinical observations might be explained by the fact that AIRE controls only a fraction of the genes promiscuously expressed in mTEC (47, 58, 59). Another fraction is controlled by the transcription factor FEZF2, which in the thymus is exclusively expressed by mTEC (59). AIRE and FEZF2 together control about 60% of the antigens promiscuously expressed in mTEC (59). Mice with a deletion of *Fezf2* in mTEC display a reduced frequency of Treg in the thymus as well as in secondary lymphoid organs, and develop a more severe autoimmune phenotype than *Aire*-deficient mice (59). In that study, 30% of mice with a deletion of *Fezf2* specifically in mTEC succumbed within 12 months from a lethal inflammatory syndrome, while all *Aire*-deficient mice survived for more than 16 months after birth (59). The lack of *Fezf2* expression in mTEC results in a different autoimmune profile than the one observed with *Aire* deficiency, leading to an infiltration of lung, liver, kidney, stomach, small intestine, salivary gland, brain, and testis but not of the pancreas and retina, which are the prominent targets in *Aire*-deficient individuals (59). Thus, FEZF2 controls the establishment of immune tolerance toward different antigens than AIRE (59, 60). The function of FEZF2 might, however, be more complex because, beyond regulating the promiscuous expression of tissue-restricted antigens in mTEC, it also influences the number and distribution of these cells within the thymic medulla (59, 60). Noteworthy, about 37% of the genes promiscuously expressed in mTEC are regulated neither by AIRE nor FEZF2, implying the existence of additional mechanisms.

Medullary thymic epithelial cells can present promiscuously expressed antigens to T cell progenitors directly or transfer these antigens to neighboring dendritic cells (DC) that can then act as antigen-presenting cells (61). These two routes of antigen presentation are required to generate the complete Treg repertoire (61). There are three major DC subsets in the thymus, resident CD8α^+^ DC and two subsets of migratory DC, namely plasmacytoid DC and Sirp1α^+^ DC that are maintained in the thymus through the migration of cells from the periphery (62–64). These three subsets rely on different chemokine receptors to reach their thymic niche. The resident *Batf3*-dependent CD8α^+^ DC express XCR1 that is the chemokine receptor for XCL1, a chemokine expressed in mTEC in an *Aire*-dependent manner (65). This chemotactic axis might promote the positioning of CD8α^+^ DC next to mTEC, and thus facilitate the capture of promiscuously expressed antigens to generate tTreg (61, 65). Sirp1α^+^ DC and pDC accumulate in the thymus in CCR2- and CCR9-dependent manners, respectively (66, 67). These two migratory DC subsets contribute to the ontogeny of tTreg since TCAF3-specific Treg develop in a DC-dependent manner and are still found in mice lacking resident CD8α^+^ DC (68). Sirp1α^+^ DC can process antigen obtained from mTEC *in vivo*, and promote Treg development *in vitro* (61, 69–72). Some pDC co-localize with Treg in the thymic medulla and can induce the development of T cell progenitors into Treg *in vitro* (71, 73). In addition to handling antigens obtained from mTEC, these migratory DC subsets might bring tissue-restricted or systemic antigens to the thymus and promote locally the formation of tTreg. Such pathway might reduce the distinction between tTreg and pTreg repertoires, as well as offer environmental antigens the possibility of contributing to tTreg selection. Importantly, the capacity of these DC to migrate to thymus, and hence to induce tTreg formation, is reduced following their stimulation *via toll*-like receptors, limiting the possibility for microbes to harness this immune suppressive axis to their advantage (63).

**Figure 1:**
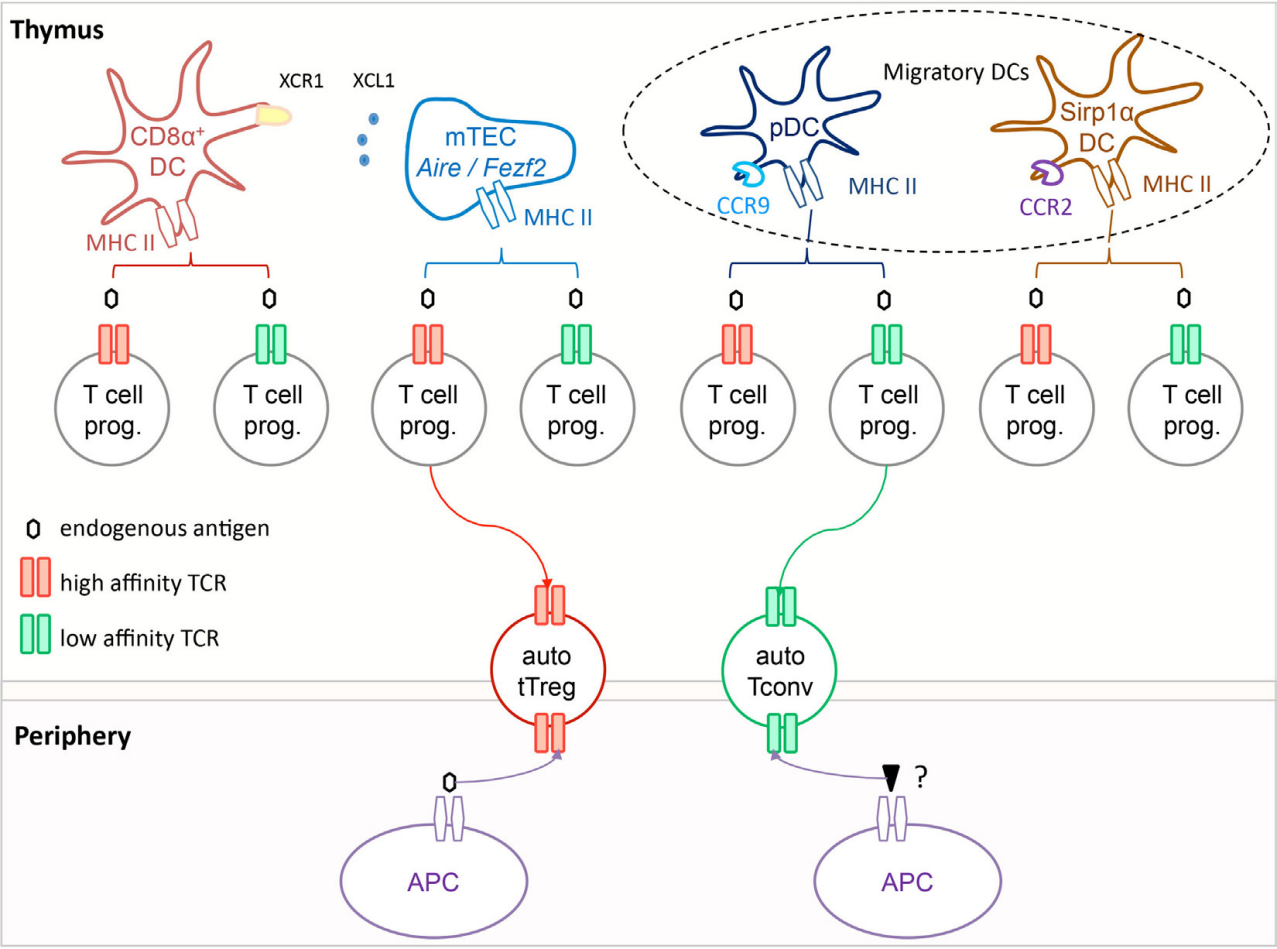
Autoreactive tTreg are selected in the thymus on self-antigens *via* T cell receptor (TCR) interactions of high functional affinity. In the thymus, T cell progenitors expressing TCR of high functional affinity (red) for self-antigens are selected into the tTreg compartment, while those expressing TCR of low functional affinity (green) are selected to become Tconv. The antigen-presenting cell can be a medullary thymic epithelial cells (mTEC), a resident CD8α^+^ dendritic cells (DC), or a migratory pDC or SIRP1α^+^ DC, or possibly a combination of these if the selection process involves multiple T cell–APC contacts. In the periphery, tTreg appear to react primarily toward the self-antigen that mediated their development, while Tconv respond most efficiently toward distinct non-self-antigens. It is noteworthy that all Tconv are positively selected, and hence have a certain degree of autoreactivity.

In sum, the available studies concur to show that autoreactive tTreg are selected on endogenous self-antigens in a highly specific manner through TCR interaction of high affinity in the thymus. Defects in the presentation of self-antigen, either due to impaired expression of this antigen or MHC polymorphism can thus create holes in the Treg repertoire. The process of tTreg selection is not monolithic: distinct molecular mechanisms control the expression and presentation of different (auto)antigens in the thymus, involving different sets of transcription factors and antigen-presenting cells (Figure 1). This raises the question of the redundancy between these pathways, and of the additional signals each of them provides to T cell progenitors besides the antigen–MHC-II complex, with the ensuing consequences this may have for T cell development and function. It is conceivable that this diversity explains the observations highlighting cases in which TCR affinity is not directly proportional to tTreg selection. Further studies of the cellular and biochemical events associated with the selection of T cell progenitors specific for distinct endogenous antigens such as MOG, TCAF3, and IRBP into tTreg might help to clarify these issues. Further investigation of these distinct pathways might also help to better understand the biochemical mechanisms that orient T cell progenitors toward either tTreg positive selection or clonal deletion when they encounter MHC-II peptide ligands of high affinity.

## How does Antigen-Specificity Contribute to Treg Suppressive Function in Human Diseases?

The suppressive activity of mature Treg involves the engagement of their TCR by antigen (25, 26). Treg require TCR–antigen–MHC-II interactions of high functional affinity to achieve significant suppressive effect in the periphery (34). Thus, autoreactive tTreg-mediated suppression may operate in a highly specific manner, due to the low degree of degeneracy in the antigen-reactivity of these cells, so that the repertoire of Treg available plays an important role for the control of immunity. A high degree of specificity would help explaining how Treg suppress unwanted immune responses, while permitting efficient defense against infectious diseases. A highly specific system, however, raises the risk of holes in the Treg repertoire, and a lack of control of some deleterious immune responses. In opposition to a high degree of specificity, it has been shown that once activated in an antigen-specific manner *via* their TCR, Treg can then suppress Tconv specific for other antigens in a bystander manner. The breadth of this bystander suppression and the mechanisms controlling its amplitude have remained imprecisely defined. There is a need to elucidate the mechanisms controlling the breadth versus specificity of Treg-mediated suppression in human diseases, which requires the possibility of following disease-relevant Treg and Tconv concomitantly in an antigen-specific manner. Recent studies have successfully provided some first insight on this key aspect of adaptive immunity.

A quantitative analysis of relevant antigen-specific Treg and Tconv was performed in Goodpasture’s disease, a rare autoimmune disorder affecting kidneys and lungs with an incidence of about one case per million individuals per year (74). A hallmark of this disease is the presence of autoantibodies against the noncollagenous domain 1 (NC1) of the α3 chain of type 4 collagen [α3(IV)NC1] (75, 76). These autoantibodies can transfer disease, as shown in monkeys infused with antibodies from patients with Goodpasture’s disease (77). Autoantibodies might cause immunopathology directly and indirectly by promoting autoaggressive T cell responses. In support of the latter, the injection of such antibodies induced disease in wild-type mice, but not in T cell-deficient mice (78). CD4^+^ T cells have direct pathogenic roles (79) and can even induce disease independently of autoantibody in rodents (80). T cells directed against α3(IV)NC1 were found in increased numbers in patients with Goodpasture’s disease during acute flares compared to control individuals (81, 82). Supporting the notion that CD4^+^ T cells are key players in this human disease, its incidence shows a strong association with HLA alleles, particularly HLA-DRB*1501 and HLA-DRB*1502 (83). Thus, about 80% of patients with Goodpasture’s disease are positive for HLA-DRB*1501 or HLA-DRB*1502, while the frequency of these alleles is only around 20% in the control population (83). By contrast, the presence of HLA-DR1 or HLA-DR7 markedly reduces the risk of disease in HLA-DRB*1501^+^ individuals (83). An important feature of this disease is that α3(IV)NC1 is the major autoantigen, so that it is possible to study the most important disease-relevant T cells by focusing only on those reacting toward this antigen. The impact of these deleterious and beneficial HLA alleles on the selection of α3(IV)NC1-reactive CD4^+^ T cells was analyzed using HLA class 2 tetramers loaded with the α3_135–145_ peptide (8). CD4^+^ T cells reacting against HLA-DR15-α3_135–145_ were present at a 100-fold higher frequency in HLA-DR15^+^ patients with Goodpasture’s disease compared to healthy HLA-DR15^+^ individuals (8). The vast majority of these cells were FOXP3^−^ and produced pro-inflammatory cytokines upon antigenic stimulation, suggesting that they represented a pathogenic population. By contrast, HLA-DR1-α3_135–145_-reactive CD4^+^ T cells were mostly FOXP3^+^CD25^hi^CD127^−^, and thus presented with a typical Treg phenotype (8). These Treg were possibly tTreg selected on α3(IV)NC1 peptides, since this protein was found to be expressed in the thymus (84). Remarkably, in HLA-DR15^+^HLA-DR1^+^ individuals, HLA-DR15-α3_135–145_-reactive CD4^+^ T cells were mostly FOXP3^−^, while HLA-DR1-α3_135–145_-reactive CD4^+^ T cells were FOXP3^+^ (8). Thus, the HLA allele associated with a higher risk of developing disease selects autoreactive Tconv, while the protective allele elicits autoreactive Treg targeting the same antigen. The relevance of this dichotomy for the development of pathology was evaluated in a model of Goodpasture’s disease using mice carrying transgenic HLA-DR15 and/or HLA-DR1 molecules on an *FcgRIIb*-deficient background. In these mice, HLA-DR1-α3_135–145_-reactive CD4^+^ T cells were, as observed in human, mostly Treg, and HLA-DR15-α3_135–145_-reactive CD4^+^ T cells were in majority Tconv. After immunization with the α3_135–145_ peptide, HLA-DR15^+^ mice developed a disease that was not affected by the prior depletion of Treg, implying a lack of protective Treg on this background (8). Other Treg were incapable of preventing disease *via* bystander suppression. By contrast, HLA-DR15^+^HLA-DR1^+^ mice remained healthy after immunization, unless Treg were depleted, demonstrating that protective Treg were restricted to HLA-DR1 (8). Thus, the HLA-DR1 allele afforded dominant protection by favoring the selection of autoreactive Treg, which counteracted the autoreactive Tconv directed against HLA-DR15-α3_135–145_. The protective function of Treg in this disease is therefore operating in a specific manner. This is striking since HLA-DR15 and HLA-DR1 differ only by few amino acids (8). These data further underline the specificity of Treg development, and of their function in the periphery. It also demonstrates that a hole in the Treg repertoire, related to the HLA profile of the individual, can lead to an unbalanced pool of autoreactive Tconv, which are selected following distinct modalities, and thus increase the susceptibility of the individual to a specific autoimmune disease. This underlines the role of HLA in the development of the Treg repertoire.

T regulatory cells are also important for limiting pathological immunity toward environmental antigens including allergens (85). IPEX patients display severe allergic manifestations including food allergy, and eczema, which are associated with increased T_H_2 responses, IgE levels, as well as eosinophilia (86, 87). Some early reports provided evidence that in young children the resolution of allergy toward cow milk products was related to the accumulation of allergen-reactive Treg (88, 89). A limitation of these studies, however, was that they only assessed Treg indirectly by comparing the proliferation of peripheral blood mononuclear cells depleted or not from CD25^+^ cells in response to an antigen stimulation, rather than by directly enumerating and characterizing antigen-reactive Treg. The direct analysis of disease-relevant Treg and Tconv can be performed using MHC-II-peptide tetramers when the antigen-specificity of these cells is known at the epitope level, and if such tetramers are available. However, the knowledge of the antigen-reactivity of disease-relevant Treg is limited in most cases. To identify disease-relevant Tconv and Treg in diseases for which the epitope landscape is not known, several laboratories developed assays that distinguish antigen-reactive Treg and Tconv according to their distinct upregulation of cell surface receptors after a short-term antigen stimulation *ex vivo* (90). A considerable asset of such methodology is that it requires neither the knowledge of the epitope(s) within the relevant antigen, nor of its MHC restriction, to make the identification of antigen-reactive cells feasible. Such an approach has recently been used to examine the antigen-reactivity of Treg and Tconv against aeroantigens, in particular birch pollen, in healthy and allergic individuals (85). This study used the fact that antigen-reactive Treg and Tconv distinctively upregulate CD137 and CD154, respectively, after a short antigen-specific stimulation of several hours (91). Although the low abundance of antigen-reactive cells makes their direct characterization based on their upregulation of these markers by flow cytometry difficult, their enrichment immediately after the stimulation by magnetic purification of CD154^+^ and CD137^+^ cells makes such cytometric analysis subsequently possible. This protocol has been used to identify, enumerate, and characterize T cells reactive toward food antigens, commensal bacteria, infectious antigens, and aeroantigens in the naïve repertoire (85, 92, 93). These analyses revealed the existence of birch-reactive Treg in the blood of healthy adults, which showed an increased proliferation during the allergen season (85). These cells were not found in cord blood, and possibly represented peripherally induced Treg. By contrast, birch-reactive Tconv did not proliferate during this season in healthy individuals, suggesting that they were efficiently regulated by Treg. Of note, the Treg expressed TCR of higher functional avidity for birch antigens than memory CD4^+^ Tconv, which possibly contributed to their dominant effect (85). Remarkably, birch-reactive Treg were present in similar numbers, and showed comparable functional properties in allergic individuals compared to the control group (85). These findings argue against the notion that allergy develops as a result of an acquired defect in the Treg compartment. However, as expected, allergic donors displayed a distinct birch-reactive Tconv profile: these cells proliferated during the allergen season and showed elevated T_H_2 cytokine expression, indicating that they escaped immune regulation (85). Treg and T_H_2 Tconv recognized different proteins within the allergen, and T_H_2 responses against a protein of the allergen were observed in an individual only when this protein was not efficiently recognized by Treg (85). This example illustrates the specificity of Treg-mediated suppression, and provides a case where it may be possible to analyze some of the factors controlling Treg-mediated bystander suppression in human. Why did Treg directed against some other proteins of the allergen not inhibit immunity toward all the proteins of this allergen? Interestingly, T_H_2 Tconv preferentially recognized water-soluble antigens rapidly released from the allergen particles when these were incubated in solution, while Treg reacted predominantly against proteins that remained associated with the non-soluble fraction of the particles (85). It is therefore possible that these distinct antigens showed a distinct bio-distribution *in vivo*, so that those generating pro-allergenic T_H_2 cells could not be subjected to Treg-mediated bystander suppression. The fact that Treg were directed against a distinguishable fraction of allergen antigens supports further the hypothesis that these were pTreg rather than tTreg. As elegantly discussed in that study, it might be possible to induce antigen-specific human pTreg by associating selected antigens with non-soluble particles alike those of allergens, which could then be delivered *via* the airways (85). Could such therapeutically induced pTreg control antigen-specific T_H_2 cells and disease in allergic patients?

## Discussion

The studies described above have underlined the importance of the composition of the Treg repertoire for the suppression of unwanted immunity in human, and hence of the mechanisms presiding at the development of this repertoire.

The positive selection of autoreactive tTreg seems to be highly specific because the removal of single endogenous antigens led to the profound loss of tTreg toward these antigens. This suggests that there is little compensation between selecting self-antigens, meaning that tTreg selected on one autoantigen usually do not recognize efficiently another autoantigen. This low degree of degeneracy could be explained by the process of negative selection, i.e., the removal of T cell progenitors that receive excessive signals during their development in the thymus. Indeed, Treg are selected by TCR–antigen interaction that are already of high affinity, close to the threshold leading to negative selection, so that the efficient recognition of additional antigens in the thymus might lead to the elimination of such cells. In support of this model, several mechanisms implicated in the selection of Treg have been shown to be important for negative selection. Thus, Aire contributes to the establishment of immune tolerance through the negative selection of autoreactive T cells. For instance, the *Aire*-dependent expression of IRBP in the thymus is necessary for the deletion of pathogenic IRBP-reactive Tconv cells that provoke autoimmune uveitis (94). The autoreactive tTreg repertoire might therefore be composed of independent units of tTreg (each unit being selected by a given autoantigen) with little inter-unit connectivity. According to this view, the size of the autoreactive tTreg repertoire is limited, at the functional level, by the number of selecting agonist peptides presented by MHC-II molecules. This number has been estimated to lie between 10^3^ and 10^5^ (39). The composition of this repertoire is also influenced by the HLA alleles expressed by the individual. It may thus be possible that the repertoire of tTreg is in some cases limited, and that some polymorphisms in HLA molecules might have some strong impact on the functional property of the tTreg repertoire by determining the absence or presence of tTreg directed toward specific antigens. This appears to be the case in Goodpasture’s disease, in which the presence of α3_135–145_-reactive Treg was dependent on the HLA-DR1 allele. Holes in the Treg repertoire create windows of opportunity for the development of deleterious immune responses. The studies described in this review validate this concept for Goodpasture’s disease and airway allergy. It is important in this context to emphasize that a hole in the Treg repertoire does not immediately lead to a pathological immune reaction. This may account for the fact that only a minority of HLA-DR15^+^HLA-DR1^−^ individuals develop Goodpasture’s disease, indicating that other mechanisms have a decisive influence on the onset of autoimmune disease, which may include other immune regulating mechanisms.

Such a high specificity in antigen-guided development and function might be unique among T cells to the Treg subset. Indeed, the development and function of Tconv appears to be more degenerate. In fact, Tconv are first selected on self-antigens in the thymus to then react against distinct foreign antigens in the periphery, implying that degenerated antigen recognition is a principle of their biology. Several studies previously attempted to estimate how many TCR can be selected in the Tconv compartment by a single positively selecting peptide using mice with restricted peptide-presenting capacity (95, 96), yet such an estimation appeared difficult to realize using such an experimental strategy (97). Other studies investigated the diversity of peptides that could positively select Tconv expressing a single TCR using fetal thymic organ culture. Testing 95 peptides selected from a set of peptides separated from I-E^k^ presented on a B cell line, Ebert and colleagues found that six could positively select CD4^+^ T cells carrying the 5C.C7 TCR, which is known to respond to a fragment of moth cytochrome *c* (MCC) in the context of I-E^k^ (98). In a separate study performed using the same strategy, Lo and colleagues found that one out of the same 95 peptides mediated the positive selection of T cell progenitors carrying the AND TCR, which also recognizes and can be activated by MCC in the periphery (99). Considering that the set of 95 peptides used in these two studies was estimated to represent 5% of the different peptide species presented by I-E^k^ molecules these findings suggest that a limited number of peptides yet more than one are able to positively select T cell progenitors expressing a given TCR into the Tconv compartment. Similar findings were obtained for CD8^+^ T cells, with the identification of two naturally occurring H-2K^b^-bound self-peptides that were able to promote the positive selection of OT-I ovalbumin-reactive CD8^+^ T cells (100). The selection of Tconv might thus be more degenerate than for Treg. There is evidence that the recognition of antigen by Tconv shows some degeneracy in the periphery, in the sense that one Tconv can react toward multiple epitopes. Some autoreactive TCR can recognize several antigens expressed in the same tissue, a situation of cumulative autoimmunity that might facilitate the activation and acquisition of effector activity by autoreactive T cells (101). The degeneracy of T cell recognition is also important during heterologous immunity, that is the contribution of memory or effector cells specific for a pathogen to the control of a distinct pathogen (102). Heterologous immunity has been observed for virus-specific CD8^+^ T cells reacting toward influenza and Epstein–Barr virus (103), and has been suggested to play a role in the development of severe acute infectious mononucleosis (104). Given this apparently higher degree of degeneracy and the higher number of Tconv present in the periphery compared to Treg (which represent only a small fraction of the T cell compartment) it seems more likely to find holes in the Treg repertoire than in the Tconv repertoire. One may speculate that this relates to the primary function of the adaptive immune system, which is to defend the host against infection.

In conclusion, the novel experimental models and technologies available to study the antigen-specificity of Treg development and function will undoubtedly lead to a great improvement of our understanding of the specificity of this vital suppressive mechanism, and illuminate how to best manipulate it therapeutically. These exciting developments are eagerly awaited.

## Author Contributions

All authors listed have made a substantial, direct, and intellectual contribution to the work and approved it for publication.

## Conflict of Interest Statement

The authors declare that the research was conducted in the absence of any commercial or financial relationships that could be construed as a potential conflict of interest.
